# Fusariosis in Mexico: cases and gaps to be addressed

**DOI:** 10.3389/fcimb.2026.1955078

**Published:** 2026-09-07

**Authors:** Ricardo Rodriguez-Vargas, Blanca Esthela López-Soliz, María Alejandra Cuen-Vázquez, Domingo Martínez-Soto

**Affiliations:** 1 Department of Microbiology, Centro de Investigación Científica y de Educación Superior de Ensenada, Ensenada, Baja California, Mexico; 2 Faculty of Medicine, Universidad Xochicalco, Ensenada, Baja California, Mexico; 3 Department of Health Sciences, Universidad de Sonora, Cd. Obregón, Sonora, Mexico

**Keywords:** fusariosis, *Fusarium*, immunocompromised patients, Mexico, opportunistic infection

## Abstract

This mini-review synthesizes four decades of Mexican fusariosis literature, organizing cases into four clinical categories: ocular, superficial/cutaneous, invasive/disseminated, and central nervous system (CNS) disease. It identified significant gaps in national surveillance, molecular diagnostics, antifungal susceptibility testing, access to effective therapies, specialized mycology infrastructure, and highlights the urgent need for a coordinated clinical mycology network in Mexico. Fusariosis, caused by *Fusarium* species, presents a broad clinical spectrum, from keratitis and onychomycosis in immunocompetent individuals to disseminated disease in those with hematologic conditions. In Mexico, most reported cases stem from clinical case reviews rather than systematic national surveillance. Exceptions include a 10-year retrospective study published in 2023, and a *Fusarium solani* meningitis outbreak in Durango and Matamoros during 2022–2023, which together represent the largest global clusters of healthcare-associated fungal meningitis including an antifungal-resistant isolate in Matamoros that required investigational treatments such as Fosmanogepix. Keratitis by *Fusarium* spp. is the most documented manifestation, with 380 cases reported, most of them in clinical centers for diagnosis in Ciudad de Mexico, where *F. solani* predominated and ocular trauma was the primary risk factor. Additionally, 49 cases of invasive fusariosis have been reported across six states, mainly linked to burn injuries and hematological malignancies. Collectively, this review provides a national framework for understanding fusariosis and informs future research and public health strategies to address this emerging threat.

## 1 Introduction

**Figure 1:**
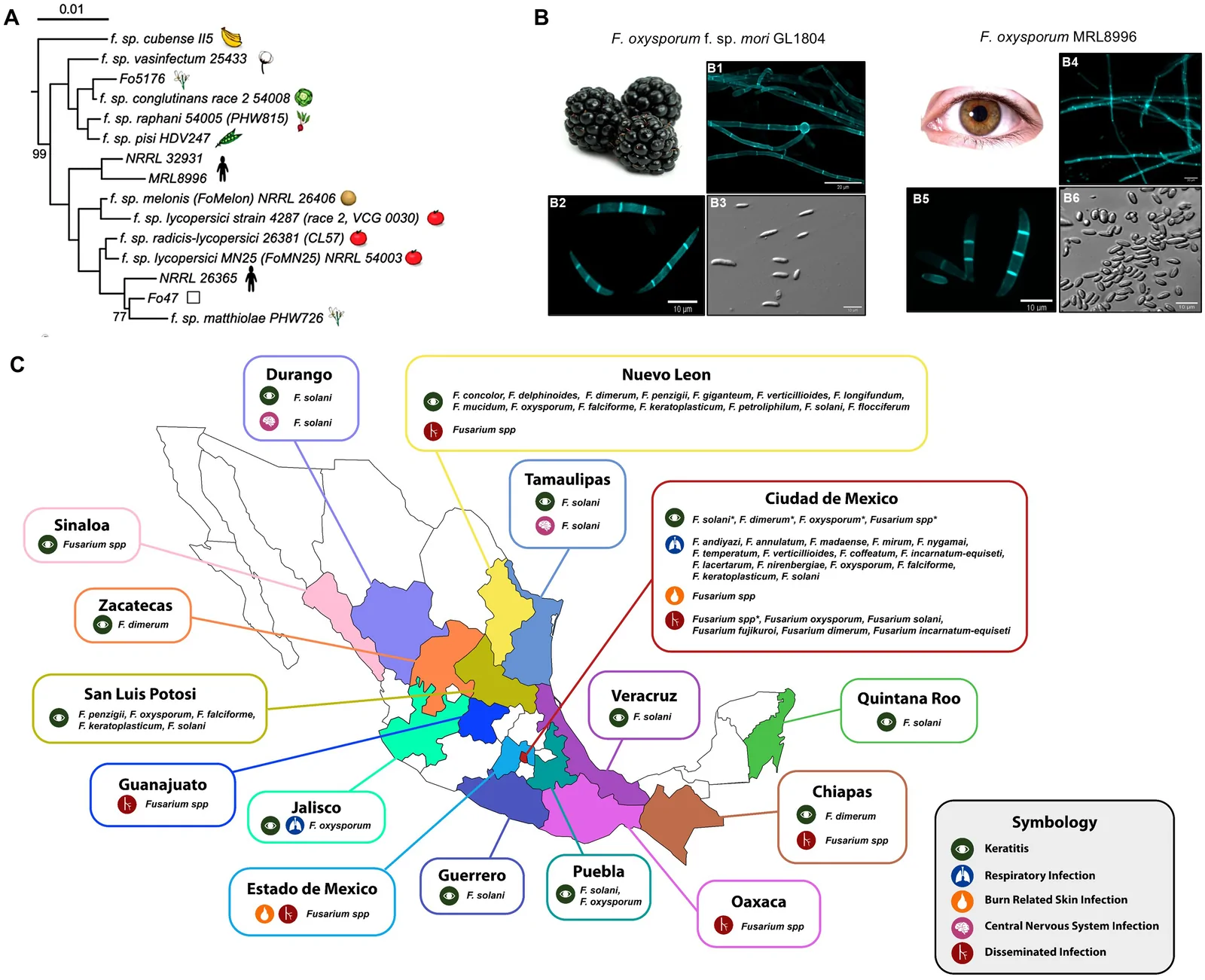
Characteristics of Fusarium oxysporum and reported cases of fusariosis in Mexico. **(A)** Phylogenetic relationships among members of the *F. oxysporum* species complex, highlighting the close genomic relatedness of human- and plant-associated lineages. Notably, human-pathogenic strains NRRL 32931 (disseminated infection), MRL8996 (keratitis), and NRRL 26365 (central nervous system infection) are polyphyletic and appear on separate branches with plant-pathogenic lineages, rather than forming a single, distinct clade. Despite differences in host range and clinical or phytopathological relevance, these taxa cluster closely within the species complex, reinforcing the need for molecular approaches to accurately identify fungal species within the *Fusarium* species complexes. Ideally, genome sequencing and comparative genomics tools would identify genes and regions specific to each pathogenic species or conserved across human pathogens that could serve as molecular markers. **(B)** Morphological comparison between the phytopathogenic *F. oxysporum* (f) sp. *mori* GL1804 and the human-pathogenic *F. oxysporum* isolate MRL8996. Fluorescence and bright-field microscopy reveal similar morphologies in both fungal pathogens. In both isolates, regardless of host (plant or human), septate mycelium **(B1, B4)**, sickle-shaped macroconidia with two to three septa **(B3, B5)**, and oval microconidia **(B3, B6)** are observed. This underscores the limited utility of conventional phenotypic features alone in distinguishing lineages with different host associations. **(C)** Map of Mexico showing the states where fusariosis cases have been documented. States with at least one reported case are highlighted. Clinical icons represent the primary tissue or syndrome associated with each report: eye (keratitis), lung (respiratory infection), artery (disseminated infection), flame (burn-related skin infection), and brain (central nervous system infection). Text boxes within each state list the reported Fusarium species. Asterisks (*) indicate cases where the *Fusarium* species was diagnosed at a clinical center located in a different state from where the sample was collected. **(A)** modified from Yu et al. (2023). https://doi.org/10.3390/jof9030359. Mycelium and macroconidia in **(B)** stained with Calcofluor white.

The genus *Fusarium* encompasses more than 300 phylogenetically distinct species grouped into approximately 23 species complexes (Bansal et al., 2019). Several of these complexes are associated with human infections, most notably the *Fusarium solani* species complex (FSSC), *F. oxysporum* species complex (FOSC), and *F. fujikuroi* species complex (FFSC) (Nucci and Anaissie, 2023). These complexes include closely related species and cryptic taxa that cannot be reliably differentiated based solely on morphological characteristics (Montoya et al., 2024; Edel-Hermann and lecomte, 2019) (**Figure 1A**). For example, regardless of lifestyle, all members of the FOSC produce septate mycelium, sickle-shaped macroconidia, and oval microconidia (Ekwomadu and Mwanza, 2023) (**Figure 1B**). Therefore, accurate molecular identification is essential for reliable diagnosis, surveillance, and epidemiological analysis.

The term “fusariosis” encompasses a heterogeneous group of infections, including onychomycosis, keratitis, cutaneous and subcutaneous diseases, fungemia, and disseminated multi organ infection (Nucci and Anaissie, 2023). The clinical significance of *Fusarium* is defined by two main features: its ability to cause life-threatening disseminated infections in immunocompromised hosts and its intrinsic resistance to most antifungal agents used in clinical practice (Al-Hatmi et al., 2016; Li et al., 2025). Among patients with hematologic malignancies, prolonged neutropenia, or hematopoietic stem cell transplantation, invasive fusariosis has mortality rates between 50-80%, rivaling or exceeding those of invasive aspergillosis in severely immunocompromised populations (Nucci et al., 2004; Muhammed et al., 2011; Sáenz et al., 2020). Consequently, *Fusarium* has recently been designated as a high-priority human fungal pathogen in global priority pathogen frameworks (Casalini et al., 2024).

In Mexico, fusariosis has historically been documented through case-specific institutional reports, particularly ophthalmologic reviews on keratitis, rather than comprehensive national epidemiological data (Villegas-Flores et al., 2012; Barragán-Reyes et al., 2023). The publication in 2023 of the first retrospective review analyzing 10 years of fusariosis cases from multiple hospitals in Mexico represented a turning point in the national understanding of this disease (Barragán-Reyes et al., 2023). Almost simultaneously, the 2022–2023 *F. solani* meningitis outbreaks linked to epidural anesthesia in Durango and Matamoros (Tamaulipas), brought international attention to fusariosis in the Mexican public health context (Strong et al., 2024).

This mini-review examines clinical evidence from all fusariosis cases reported in Mexico over the last four decades. It draws on original articles and official reports published in English or Spanish, identified through PubMed, Google Scholar, EBSCO, and Mexican university repositories. The review classifies cases by clinical manifestation, analyzes geographic and diagnostic patterns, and highlights gaps in surveillance, fungal identification methods, reporting, and antifungal management. It provides a structured framework for understanding the national landscape of fusariosis and aims to inform future research and public health strategies addressing this emerging threat.

## 2 Clinical relevance of fusariosis

The clinical presentation of fusariosis is determined primarily by the immune status of the host and the route of inoculation (Nucci and Anaissie, 2007). In immunocompetent individuals, *Fusarium* most frequently causes onychomycosis and keratitis following corneal trauma, particularly in agricultural settings (Mohamed-Noriega et al., 2026; Cighir et al., 2023). Occasionally, localized cutaneous or subcutaneous infections occur after traumatic inoculation or burn injury (Gonzalez Guerrero et al., 2025). These forms are rarely life-threatening and, when adequately treated, generally respond to antifungal therapy, though relapse or treatment failure may occur in a subset of patients (Dignani and Anaissie, 2004).

Immunocompromised hosts face a different prognosis. In patients with neutropenia, hematologic malignancy (Batista et al., 2020), or solid organ or hematopoietic stem cell transplantation, *Fusarium* can breach mucosal or cutaneous barriers, disseminate hematogenously, and produce the characteristic clinical triad of invasive fusariosis: persistent fever unresponsive to broad-spectrum antibacterials, mold-positive blood cultures, and distinctive targetoid or necrotic cutaneous lesions at distant sites (Nucci et al., 2004; Nucci and Anaissie, 2007). The capacity for true fungemia makes *Fusarium* easily detectable via blood culture, a valuable diagnostic advantage that should be prioritized in clinical practice (Lockwood and Crescencio, 2015).

From a therapeutic perspective, *Fusarium* poses significant challenges. The genus is intrinsically resistant to fluconazole, echinocandins, and terbinafine, and exhibits heterogeneous susceptibility to voriconazole and amphotericin B, with responses varying widely among species complexes and even among isolates within a complex (**Table 1**) (Al-Hatmi et al., 2016; Strong et al., 2024; Li et al., 2025; Campos-Jiménez et al., 2026). The *Fusarium solani* species complex (FSSC), responsible for most invasive cases worldwide and the predominant agent in Mexican reports, displays high levels of intrinsic antifungal resistance among clinically relevant *Fusarium* taxa (Al-Hatmi et al., 2016; Barragán-Reyes et al., 2023; Fungicide Resistance Action Committee (FRAC), 2026). Another important *Fusarium* species reported in Mexico is *F. oxysporum*, which also exhibits high levels of intrinsic antifungal resistance (reviewed by Campos-Jiménez et al., 2026).

**Table 1:** Epidemiological data and antifungal treatments reported in Mexico to treat cases of fusariosis.

Infection type	Gender and age	Species	State	Clinical center for diagnosis	Method of diagnosis	Antifungal(s) used	*FRAC classification	Reference
Ocular (keratitis)	61 cases: 78.7% male with average age of 41.5 years old and 21.3% female with average age of 40 years old	*F. solani, F. dimerum, F. oxysporum, Fusarium* spp.	Ciudad de México	Asociación para Evitar la Ceguera en México (APEC)/Hospital “Luis Sánchez Bulnes”	Corneal scraping cultured on selective medium, and morphological identification by microculture techniques and microscopic examination	Combined therapy of natamycin, ketoconazole, miconazole, itraconazole, fluconazole.	Group 48 F8: ergosterol binding (natamycin). Not listed by FRAC mechanism of action Group 3, G1: C14-demethylase in sterol biosynthesis (ketoconazole, miconazole, itraconazole, fluconazole)	Pérez-Balbuena et al., 2009
	77 cases: 75.3% male and 24.6% female the average age was 46 years old					Topical natamycin and ketoconazole	Group 48 F8: ergosterol binding (natamycin). Not listed by FRAC mechanism of action Group 3, G1: C14-demethylase in sterol biosynthesis (ketoconazole)	Vanzzini Zago et al., 2010
	100 cases: 66% male and 34% female the average age was 48 years old	*F. solani*				Topical natamycin and voriconazole	Group 48 F8: ergosterol binding. (natamycin) Not listed by FRAC mechanism of action Group 3 G1: C14-demethylase in sterol biosynthesis (voriconazole)	Urcullo-Terrazas et al., 2017
	Cases with different ages: Males: 7, 28, 41, 47, 49, 76 years old. Females: 32, 34, 75 years old.	*F. solani*	Puebla, Quintana Roo, Ciudad de México, Veracruz, Tamaulipas, Durango, Guerrero		Ocular scraping culture on Sabouraud Dextrose Emmons agar, followed by morphological identification using microculture techniques and microscopic examination	Not mentioned	–	Meza-Menchaca et al., 2020
	Male of 73 years old Females: 30, 41 years old	*F. dimerum*	Zacatecas, Chiapas, Ciudad de México					
	Male of 62 years old	*F. oxysporum*	Puebla					
Ocular (keratitis)/respiratory infections	Not specified	*F. concolor* *F. delphinoides* *F. dimerum* *F. penzigii* *F. giganteum* *F. verticillioides* *F. longifundum* *F. mucidum* *F. oxysporum* *F. falciforme* *F. keratoplasticum* *F. petroliphilum* *F. solani* *F. flocciferum*	Nuevo León	Not mentioned	Phenotypic and molecular identification (fungal culture and morphological examination under microscopy, PCR and sequencing of ITS/TEF1-α/RPB2/calmodulin).	Antifungal susceptibility was tested; the list of antifungals was not used as treatment: Amphotericin, fluconazole, itraconazole, ketoconazole, isavuconazole, posaconzole, voriconazole, anidulafungin, caspofungin, micofungin, 5-flucytosin, difoconzole, propioconazole, tebuconazole.	Group 48 F8: ergosterol binding (amphotericin) Group 8 G4: squalene epoxidase in sterol biosynthesis (erg1) (terbinafine) Not listed by FRAC mechanism of action Group 3 G1: C14-demethylase in sterol biosynthesis (fluconazole, itraconazole, ketoconazole, isavuconazole, posaconzole, voriconazole, difenconazole, propiconazole, tebuconazole). Not listed by FRAC (anidulafungin, caspofungin, micofungin, 5-flucytosin)	Montoya et al., 2024
		*F. andiyazi* *F. annulatum* *F. madaense* *F. mirum* *F. nygamai* *F. temperatum* *F. verticillioides* *F. coffeatum* *F. incarnatum-equiseti* *F. lacertarum* *F. nirenbergiae* *F. oxysporum* *F. falciforme* *F. keratoplasticum* *F. solani*	Ciudad de México					
		*F. penzigii* *F. oxysporum* *F. falciforme* *F. keratoplasticum* *F. solani*	San Luis Potosi					
		*F. oxysporum*	Jalisco					
Ocular (keratitis)	10-year-old, female	*Fusarium* spp.	Sinaloa	CODET Vision Institute and Hospital General de Tijuana, Mexico.	Fungal culture on Sabouraud Dextrose and blood agar, followed by morphological identification under microscopy	Intravenous voriconazole and topical natamycin for seven days prior to surgery and an additional 6 days after keratoplasty. Topical natamycin continued for 3 weeks.	Not listed by FRAC mechanism of action Group 3 G1: C14-demethylase in sterol biosynthesis (voriconazole) Group 48 F8: ergosterol binding (natamycin)	Chacon-Cruz et al., 2019
Burn related skin infection	2 males: 18 and 19 years old. 2 females: 20 and 66 years old		Ciudad de México	Centro Nacional de Investigación y Atención al Quemado (CENIAQ), Instituto Nacional de Rehabilitación “Luis Guillermo Ibarra Ibarra”	Skin biopsy and fungal culture on Sabouraud agar.	Amphotericin B deoxycholate	Group 48 F8: ergosterol binding (amphotericin B)	Carrillo-Esper et al., 2017
Disseminated infection/Burn related skin infection	49 patients: 53% males and 47 females average age was 26 years old		Ciudad de México, Chiapas, Estado de México, Nuevo León, Guanajuato, Oaxaca	Ciudad de México: Instituto Nacional de Cancerología, Instituto Nacional de Rehabilitación “Luis Guillermo Ibarra Ibarra”, Hospital Médica Sur. Chiapas: Hospital de Especialidades Pediátricas. Estado de México: Hospital para el Niño, Instituto Materno Infantil del Estado de México. Nuevo León: Hospital Universitario “Dr. José Eleuterio González” (UANL, Monterrey). Guanajuato: Hospital Regional de Alta Especialidad del Bajío. Oaxaca: Hospital Regional de Alta Especialidad de Oaxaca.	Fungal morphological identification and one case by MALDI-TOF MS (matrix-assisted laser desorption/ionization time-of-flight mass spectrometry)	Voriconazole (30%), conventional amphotericin B (16%), liposomal amphotericin B formulations (10%) monotherapy in 67% total; combination therapy: triazole + lipid amphotericin B	Not listed by FRAC mechanism of action Group 3 G1: C14-demethylase in sterol biosynthesis. (voriconazole, triazole) Group 48 F8: ergosterol binding(amphotericin B)	Barragán-Reyes et al., 2023
Disseminated infection	33 patients: 54.5% males and 42.4 females average age was 6 years old		Nuevo León	Hospital Universitario “Dr. José Eleuterio González” (UANL, Monterrey)	Skin biopsy and nasal mucosa biopsy	Combined liposomal amphotericin B (L-AmB) and voriconazole	Group 48 F8: ergosterol binding. (amphotericin B) Not listed by FRAC mechanism of action Group 3 G1: C14-demethylase in sterol biosynthesis (voriconazole)	Vaquera-Aparicio et al., 2025
	Male of 30 years old		Ciudad de México	Centro Médico Nacional 20 de Noviembre, Instituto de Seguridad y Servicios Sociales para los Trabajadores del Estado (ISSSTE)	Skin biopsy and fungal culture			Gomez Gonzalez et al., 2025
	n=19 patients 11 males with age of 51.5 ± 17.9 years The 8 females age are not specified in text.	*Fusarium oxysporum*, *Fusariun solani*, *Fusariun fujikuroi*, *Fusarium dimerum*, *Fusarium incarnatum-equiseti*		Clinical Microbiology Laboratory of the Instituto Nacional de Ciencias Médicas y Nutrición Salvador Zubirán	Biopsy and fungal cultures on Sabouraud agar, and amplification and sequencing of the translation elongation factor-1α gene (EF1α).	Combined liposomal amphotericin B (L-AmB) with alternate use of voriconazole and itraconazole	Group 48 F8: ergosterol binding. (amphotericin) Not listed by FRAC mechanism of action Group 3 G1: C14-demethylase in sterol biosynthesis (voriconazole, itrsconazole)	Román-Montes et al., 2025
Central nervous system	80 cases: 95% females and 5% males with a average age of 30 years old	*F. solani*	Durango	Four healthcare facilities (not individually named)	Brain biopsy culture on blood agar, lactophenol blue microscopy observations, MALDI-TOF mass spectrometry, and EF1α Sanger sequencing. Fungal quantification using qPCR in samples of cerebrospinal fluid (CSF).	Liposomal amphotericin B (AmBisome) + voriconazole	Group 48 F8: ergosterol binding. (amphotericin) Not listed by FRAC mechanism of action Group 3 G1: C14-demethylase in sterol biosynthesis (voriconazole)	García-Rodríguez et al., 2024
	Not reported Male and female patients		Tamaulipas	Two private ambulatory surgical facilities (not individually named)	Detection of β-D-glucan on CSF, fungal culture, detection and quantification by PCR/qPCR, and metagenomic sequencing	Triple therapy with liposomal amphotericin B intravenous voriconazole, and fosmanogepix	Group 48 F8: ergosterol binding. (amphotericin) Not listed by FRAC mechanism of action Group 3 G1: C14-demethylase in sterol biosynthesis (voriconazole) Not listed by FRAC (fosmanofepix)	Smith et al., 2024

*Fungicide Resistance Action Committee (FRAC). (2026). FRAC Code List 2026 available at: https://www.frac.info/media/s1zfrjqa/frac-code-list-2026.pdf.

## 3 Fusariosis in Mexico

Documentation of fusariosis in Mexico spans more than four decades, with the earliest traceable cases dating to the 1980s and extending through the major nosocomial outbreaks reported in 2022–2023. The published literature can be organized into four clinical categories that reflect distinct host immune states, modes of infection, and therapeutic challenges: *i*) ocular fusariosis, *ii*) superficial and cutaneous disease, *iii*) invasive and disseminated fusariosis, and *iv*) central nervous system (CNS) infection. Unfortunately, the distribution of reported cases is influenced more by the availability of specialized diagnostic capabilities and clinical center for diagnosis (**Table 1**) than by the pathogen’s actual environmental presence or true epidemiological distribution.

### 3.1 Ocular fusariosis

Ocular infection represents the most documented clinical form of fusariosis in Mexico, with keratitis accounting for most cases. Three independent reviews from over three decades were published by the Asociacion para Evitar la Ceguera en Mexico (APEC) in Ciudad de Mexico, collectively documenting approximately 380 cases of *Fusarium* keratitis from 1981 to 2016 (Pérez-Balbuena et al., 2009; Vanzzini Zago et al., 2010; Urcullo-Terrazas et al., 2017). In all reviews, *F. solani* was consistently identified as the predominant causal agent. Ocular trauma from vegetative matter was the leading predisposing event, documented in 36–57% of patients, and agricultural occupation was a recurring risk factor, findings consistent with the global epidemiology of *Fusarium* keratitis in subtropical settings (Brown et al., 2021).

Medical therapy with topical natamycin or voriconazole resulted in resolution in approximately 45–80% of patients across these reviews; the remainder required surgical intervention, with penetrating keratoplasty performed in 23–37% of cases and evisceration necessary in 8–23% of those with most advanced disease (Pérez-Balbuena et al., 2009; Vanzzini Zago et al., 2010; Urcullo-Terrazas et al., 2017). The concentration of documented cases at a single referral ophthalmology center in Ciudad de Mexico reflects surveillance bias, given the environmental ubiquity of *Fusarium* spores and the large agricultural workforce in rural areas and Mexican states far from the capital. It is likely that several clinically relevant cases of keratitis occur beyond the reach of this institution and have not been documented. Additionally, a case of endogenous *Fusarium* endophthalmitis associated with preexisting onychomycosis in a diabetic patient was reported in Monterrey (Nuevo Leon), highlighting the potential for superficial *Fusarium* infections to serve as reservoirs for invasive ocular disease (Tamez-Peña et al., 2010). In a related study, Meza-Menchaca et al. (2020) tested 13 corneal *Fusarium* isolates from non-immunocompromised keratitis patients from APEC; these isolates came from patients registered across nine states of Mexico and were identified mainly as *F. solani*, *F. dimerum*, and *F. oxysporum* (**Table 1**). These ocular isolates demonstrated cross-kingdom infectivity by colonizing plant tissue, consistent with their environmental, plant-associated origins. The same isolates were also used to inoculate human nail tissue *ex vivo*. This experimental back-infection supports the Monterrey endophthalmitis case, suggesting that superficial keratinized tissues such as nails and cornea may act as reservoirs for the *Fusarium* strains.

### 3.2 Superficial and cutaneous fusariosis

Superficial fusariosis, predominantly onychomycosis, has been documented in Mexico primarily through case reports and small reviews from the dermatology and mycology services of the Hospital General de Mexico (HGM) (**Table 1**). The clinical spectrum includes fingernail and toenail onychomycosis, paronychia, and interdigital maceration, most often caused by *F. solani* in patients with diabetes mellitus or local predisposing factors (Herrán et al., 2003; Martinez-Hernandez et al., 2014). A study at the HGM screened 68 patients with hematological malignancies specifically for *Fusarium* onychomycosis, documenting a prevalence of 1.5%, a finding of clinical significance because *Fusarium* nail infection is a recognized reservoir for hematogenous dissemination in neutropenic patients (Araiza et al., 2020).

Burn wound infection represents the most clinically severe form of cutaneous fusariosis. A reviews from the Centro Nacional para la Atencion de las Personas con Discapacidad (CENIAQ) documented four patients with thermal burn injuries covering 30–70% of body surface area who developed cutaneous fusariosis between 2014 and 2016, they were treated with amphotericin B deoxycholate as primary therapy (Carrillo-Esper et al., 2017). These cases constitute the first systematic description of *Fusarium* burn wound infection in Mexico and highlight the susceptibility of burn patients with disrupted cutaneous barrier integrity, immune dysregulation, and prolonged hospitalization to environmental *Fusarium* acquisition.

### 3.3 Invasive and disseminated fusariosis

The most comprehensive report of invasive fusariosis in Mexico is the study of Barragán-Reyes et al. (2023), which enrolled 49 patients with confirmed fusariosis diagnosed between 2010 and 2019 at eight hospitals across six states (Ciudad de Mexico, Chiapas, Estado de Mexico, Nuevo Leon, Guanajuato, and Oaxaca) (**Figure 1C**). The principal health issues associated with fusariosis were burn injury (49%), hematological malignancies like acute leukemia (37%). Skin and soft tissue were the most frequently involved anatomical site (49% of cases), followed by fungemia (8%) and pulmonary involvement. Antifungal monotherapy was used in 67% of patients, most often voriconazole or amphotericin B formulations, and combination therapy in 20%. The overall fusariosis mortality was 22%, with higher rates in disseminated disease and delayed-treatment cases.

Subsequent reports further define the spectrum of invasive fusariosis in Mexico. A 2025 case report documented disseminated fusariosis in a 30-year-old male with acute lymphoblastic leukemia and severe neutropenia, a whom cutaneous lesions served as the diagnostic clue to fungemia, managed with combined liposomal amphotericin B (L-AmB) and voriconazole (Gomez Gonzalez et al., 2025). In pediatric population, the first reported cases of invasive fungal infections among children were at the Hospital Universitario “Dr. José Eleuterio González” (UANL, Monterrey) involved 33 patients from 2018 to 2024, with *Aspergillus* spp. and *Fusarium* spp. as the dominant fungal pathogens. The health issues predominantly associated were leukemias, with mortality of 21.2% due to invasive fungal infection (Vaquera-Aparicio et al., 2025).

### 3.4 Central nervous system fusariosis

The most unprecedented cases of fusariosis in Mexico and worldwide was the occurrence of two simultaneous healthcare-associated *F. solani* meningitis outbreaks in 2022–2023, both linked to contamination of neuraxial anesthetic solutions. These outbreaks redefined the national understanding of fusariosis by demonstrating that immunocompetent individuals can develop life-threatening CNS infection in the absence of any recognized predisposing condition, solely through iatrogenic exposure during routine surgical procedures.

The fusariosis outbreak in Durango, Mexico (May 2022 – February 2023) involved 80 patients who developed *F. solani* meningitis after epidural or spinal anesthesia at four healthcare facilities, including both public and private-sector hospitals (García-Rodríguez et al., 2024). Ninety-five percent of patients were women, 76.3% had undergone cesarean delivery, and none had recognized immunodeficiency. Contamination is suspected to have occurred in bupivacaine solutions. Clinical symptoms included severe headache, photophobia, meningismus, and cranial nerve palsies appearing 1–4 weeks post-procedure. Cerebrospinal fluid analysis revealed elevated protein levels, moderate pleocytosis, elevated 1→3-β-D-glucan levels, and a positive *Fusarium* culture. Treatment with combined voriconazole and amphotericin B was initiated in 88.8% of patients, and earlier initiation of therapy within five days of hospitalization was associated with significantly better survival (hazard ratio for delayed treatment 2.1; p < 0.05). Total mortality was 51.3%, establishing this as the most lethal recorded cluster of healthcare-associated fungal meningitis in the world literature (García-Rodríguez et al., 2024).

The fusariosis outbreak in Matamoros (Tamaulipas) in 2023 involved patients, including U.S. residents who had traveled to Mexico for elective cosmetic procedures and were exposed to epidural anesthesia at two private ambulatory surgical facilities. The Centers for Disease Control and Prevention (CDC) identified 185 American residents in at least 22 states of the United States who might be at risk of fungal meningitis because they received epidural anesthesia at the clinics of interest in 2023. Among these patients, there were 11 suspected, 10 probable, and 10 confirmed cases, with an undetermined number of additional Mexican patients (Smith et al., 2024; Chiu et al., 2025). The Matamoros outbreak differed critically from the Durango cases in one decisive respect: the causative *F. solani* isolate demonstrated resistance to all commercially available antifungals, including voriconazole and all lipid formulations of amphotericin B, with minimum inhibitory concentrations exceeding epidemiological cutoff values for all tested agents (Strong et al., 2024; Smith et al., 2024). This resistance profile rendered standard-of-care therapy ineffective, prompting emergency compassionate use of fosmanogepix, an investigational glycosylphosphatidylinositol (GPI) anchor synthesis inhibitor targeting the Gwt1 enzyme, which retains activity against polyene- and azole-resistant *Fusarium* strains. Of the 13 patients reported by Strong, et al., 2024 nine died (mortality ~69%), while three of the four survivors had received fosmanogepix monotherapy. Overall estimated mortality across the full Matamoros outbreak exceeded 50% (Smith et al., 2024). Both outbreaks were reviewed by Strong and Ostrosky-Zeichner (2024), who identified the absence of validated clinical breakpoints for *Fusarium* and the limited CNS pharmacokinetic penetration of available agents as principal therapeutic barriers.

## 4 Geographic distribution and surveillance bias

The geographic distribution of documented fusariosis in Mexico (**Figure 1C**) highlights the uneven availability of specialized diagnostic and clinical infrastructure, rather than reflecting the true epidemiological boundaries of the pathogen. Ocular fusariosis is concentrated at ophthalmology referral centers in Ciudad de Mexico, primarily APEC and, to a lesser extent, at centers in Monterrey (Nuevo Leon). Superficial fusariosis has been documented mainly by dermatology services at the Hospital General de Mexico. Invasive and disseminated fusariosis mirrors the geographic reach of specialized onco-hematology and burn care, with documented cases in the six-state corridor of Ciudad de Mexico, Chiapas, Estado de Mexico, Nuevo Leon, Guanajuato, and Oaxaca, as well as in pediatric oncology services in Monterrey (Barragán-Reyes et al., 2023; Vaquera-Aparicio et al., 2025). The 2022–2023 CNS outbreaks emerged from Durango and Tamaulipas, states not previously represented in the national fusariosis literature. Beyond the clinical reports, multicentric species distribution data support the notion that *Fusarium* is more broadly established in Mexico than published fusariosis cases alone suggest. A study by Montoya et al. (2024) reported 116 clinical and environmental *Fusarium* isolates from Nuevo Leon, Ciudad de Mexico, Guanajuato, Jalisco, and San Luis Potosí (**Figure 1C**), documenting 27 species across eight species complexes and identifying 11 taxa not previously reported in Mexico (**Table 1**). Seven of these species (*F. coffeatum*, *F. flocciferum*, *F. giganteum*, *F. longifundum*, *F. madaense*, *F. mucidum*, and *F. mirum*) were newly associated with respiratory and keratitis samples. This highlights that clinically relevant *Fusarium* diversity extends beyond the species complexes typically identified in routine diagnostics. These findings highlight that the observed geographic and taxonomic concentration of documented fusariosis is largely determined by the presence of mycology services. Most Mexican states lack specialized mycology laboratories, mandatory notification systems, or published clinical reviews, despite the prevalence of *Fusarium* in soil environments posing ongoing risks. At-risk populations include agricultural workers exposed to ocular and cutaneous inoculation, as well as patients receiving oncological or transplant care at regional hospitals where institutional mycology capacity is limited (Corzo-León et al., 2015).

## 5 Critical gaps and priorities

The published literature identifies several gaps representing urgent priorities for improving the study of fusariosis in Mexico. First, the absence of a national surveillance system for invasive fungal infections makes it impossible to estimate national incidence, track trends, or detect outbreaks before they reach the scale seen in Durango and Matamoros (Strong et al., 2024; Smith et al., 2024). Fusariosis should be considered on Mexico list of notifiable infections, with mandatory reporting by mycology and clinical laboratories at national referral centers.

Second, diagnostic capacity remains inconsistent. As summarized in **Table 1**, most reported cases have been diagnosed via direct microscopy, culture, and morphological identification, while only a limited number of studies have employed molecular or analytical methods. This indicates that species-level identification and antifungal susceptibility testing are not routinely available and are largely restricted to specialized laboratories or implemented only during epidemiological outbreaks. Expanding access to molecular identification, including sequencing of informative loci such as the D1/D2 region of the nuclear large subunit rRNA gene (*LSU*) (Zhang et al., 2006), *TEF1α* and *RPB2*, alongside susceptibility testing would enhance fungal species resolution, therapeutic decision-making, and antifungal-resistance surveillance (Thomas et al., 2019; Bugingo et al., 2025; Fungicide Resistance Action Committee (FRAC), 2026). Even more, accurate molecular identification using multiple molecular markers or, ideally, specific genes and genomic regions of human-pathogenic *Fusarium* species, identified through comprehensive comparative genomic analyses, would be essential for reliable diagnosis, surveillance, and epidemiological interpretation.

Third, effective antifungal treatments for fusariosis in Mexico remain limited. Both voriconazole and lipid formulations of amphotericin B are available at major referral centers, but access is inequitable at regional hospitals and within the public healthcare sector. Investigational agents such as fosmanogepix, which demonstrated critical utility in the Matamoros outbreak, are not commercially available in Mexico (Smith et al., 2024). Ensuring access to clinical trials of novel antifungals against resistant *Fusarium* strains should be considered a national priority.

## 6 Conclusions

The documented history of fusariosis in Mexico reveals a broad range of cases, extending from isolated keratitis and onychomycosis at referral centers to internationally recognized outbreaks of invasive and healthcare-associated Fusarium infections. The 2022–2023 CNS outbreaks notably demonstrated that severe fusariosis can occur even in immunocompetent individuals when contaminated medical products are implicated, underscoring the significant public health risk posed by resistant strains in environments lacking robust mycological surveillance. *F. solani* has consistently emerged as the predominant causative agent across various clinical infections, including keratitis, nail infections, disseminated disease, and fungal meningitis, solidifying its role as the principal clinically relevant species complex in Mexico. However, other emerging threats, such as the *Fusarium oxysporum* species complex, also require close monitoring and enhanced surveillance due to their pathogenic potential and variable antifungal susceptibility profiles.

Addressing the surveillance, diagnostic, and therapeutic gaps identified in this review will require a coordinated national response. Essential steps include instituting mandatory reporting of fusariosis, adopting standardized molecular identification and antifungal susceptibility testing, ensuring equitable access to effective antifungal therapies, and establishing a national clinical mycology network to systematically collect data from healthcare institutions across all Mexican states. In the absence of these measures, published reports will remain limited to what referral centers detect, failing to capture the true nationwide burden of *Fusarium* infections or to identify emerging resistant lineages in time.
